# Prediction Scores Identifying Patients at High Risk of Endocarditis in Enterococcal Bacteremia

**DOI:** 10.1093/ofid/ofaf796

**Published:** 2025-12-23

**Authors:** Virgile Zimmermann, Nicolas Fourré, Bruno Ledergerber, Jana Epprecht, Pierre Monney, Georgios Tzimas, Michelle Frank, Laurence Senn, Nicoleta Ianculescu, Lars Niclauss, Matthias Kirsch, Mathias Van Hemelrijck, Omer Dzemali, Benoit Guery, Barbara Hasse, Matthaios Papadimitriou-Olivgeris

**Affiliations:** Infectious Diseases Service, Lausanne University Hospital and University of Lausanne, Lausanne, Switzerland; Infectious Diseases Service, Lausanne University Hospital and University of Lausanne, Lausanne, Switzerland; Department of Infectious Diseases and Hospital Epidemiology, University Hospital Zurich and University of Zurich, Zurich, Switzerland; Department of Infectious Diseases and Hospital Epidemiology, University Hospital Zurich and University of Zurich, Zurich, Switzerland; Department of Cardiology, Lausanne University Hospital and University of Lausanne, Lausanne, Switzerland; Department of Cardiology, Lausanne University Hospital and University of Lausanne, Lausanne, Switzerland; Department of Cardiology, University Hospital Zurich and University of Zurich, Zurich, Switzerland; Infectious Diseases Service, Lausanne University Hospital and University of Lausanne, Lausanne, Switzerland; Infection Prevention and Control Unit, Lausanne University Hospital and University of Lausanne, Lausanne, Switzerland; Department of Cardiology, Lausanne University Hospital and University of Lausanne, Lausanne, Switzerland; Department of Cardiac Surgery, Lausanne University Hospital and University of Lausanne, Lausanne, Switzerland; Department of Cardiac Surgery, Lausanne University Hospital and University of Lausanne, Lausanne, Switzerland; Department of Cardiac Surgery, University Hospital Zurich and University of Zurich, Zurich, Switzerland; Department of Cardiac Surgery, University Hospital Zurich and University of Zurich, Zurich, Switzerland; Department of Cardiac Surgery, City Hospital of Zurich—Triemli, Zurich, Switzerland; Center for Experimental and Translational Cardiology, University of Zurich, Zurich, Switzerland; Infectious Diseases Service, Lausanne University Hospital and University of Lausanne, Lausanne, Switzerland; Department of Infectious Diseases and Hospital Epidemiology, University Hospital Zurich and University of Zurich, Zurich, Switzerland; Infectious Diseases Service, Lausanne University Hospital and University of Lausanne, Lausanne, Switzerland; Infectious Diseases Service, Hospital of Valais and Institut Central des Hôpitaux, Sion, Switzerland

**Keywords:** enterococci, *Enterococcus faecalis*, infective endocarditis, NOVA score, predictive score

## Abstract

**Background:**

Clinical prediction scores such as NOVA and DENOVA aim to identify patients with enterococcal bacteremia at low risk of infective endocarditis (IE) in whom imaging might be safely avoided. The aim was to evaluate the performance of NOVA and DENOVA scores and to introduce a modified tool, DENOVi.

**Method:**

This retrospective study included adult patients with enterococcal bacteremia at 2 Swiss tertiary centers (2015–2024). IE was adjudicated by multidisciplinary Endocarditis Teams according to 2023 Duke-International Society of Cardiovascular Infectious Diseases criteria. Patients were stratified as high risk for IE using the adapted NOVA score (cutoff: ≥4), the DENOVA score (≥3), and a newly developed DENOVi score (≥2), which excluded the subjective murmur criterion and broadened “valve disease” to include intracardiac electronic devices (new Vi component).

**Results:**

Among 827 bacteremia episodes, 172 (21%) were diagnosed with IE. The adapted NOVA, DENOVA, and DENOVi scores classified 76%, 26%, and 42% of patients as high risk, respectively. Corresponding NLRs were 0.04 (95% CI, .01–.15), 0.10 (0.06–0.16), and 0.04 (0.02–0.10). The adapted NOVA substantially increased the proportion of echocardiograms needed to be performed from 58% based on clinical evaluation alone to 76%, whereas the DENOVA and DENOVi scores would have reduced this proportion to 26% and 42% of episodes, respectively.

**Conclusions:**

Both adapted NOVA and DENOVi scores reliably ruled out IE, but DENOVi provided the most balanced approach between diagnostic safety and resource utilization. DENOVi therefore represents a pragmatic and objective tool for IE risk stratification in enterococcal bacteremia. Prospective validation is warranted.

## Teaser

Among 827 episodes of enterococcal bacteremia, the adapted NOVA and DENOVi scores demonstrated low negative likelihood ratios (0.04), compared with DENOVA (0.10). The adapted NOVA, DENOVA, and DENOVi scores classified 76%, 26%, and 42% of patients as high risk, respectively.

Enterococci, particularly *Enterococcus faecalis*, are among the leading bacterial causes of infective endocarditis (IE) [1–3]. IE due to enterococci is associated with substantial morbidity and mortality, especially in older patients and in individuals with predisposing cardiac conditions or implanted devices. With the rising prevalence of intracardiac electronic devices and transcatheter valve interventions, the incidence of enterococcal IE has further increased [1, 4]. This is partly attributable to the fact that these procedures are predominantly performed in elderly patients, who are more prone of enterococcal IE [5]. Additionally, the majority of transcatheter aortic valve implantations are performed via the femoral approach, a site frequently colonized by enterococci, combined with the routine use of cephalosporins for periprocedural prophylaxis, which lack activity against enterococci [6, 7]. According to the 2023 European Society of Cardiology (ESC) guidelines, echocardiographic evaluation is recommended for all patients with *E. faecalis* bacteremia [8, 9]. However, this strategy, while safe, imposes a considerable burden on healthcare resources.

To address this challenge, clinical prediction scores such as NOVA and DENOVA have been proposed to help identify patients at high risk of IE [10, 11]. The NOVA score, which includes 4 variables each weighted between 1 and 5 points, provides excellent sensitivity, with a threshold of ≥4 points effectively identifying patients at high risk for IE [10, 12, 13]. However, its low specificity means that a large proportion of patients are classified as high risk, even when IE is unlikely [10, 12, 13]. A modified version of the NOVA score was proposed for cases where only 2 sets of blood cultures [13], in which 2 out of 2 positive cultures fulfill the “N” component of the score [10, 13]. The DENOVA score was developed specifically for patients with monomicrobial *E. faecalis* bacteremia and includes 6 weighted variables [11]. Using a threshold of ≥3 points, DENOVA maintains good sensitivity while offering improved specificity over the NOVA score [11, 12, 14]. This enhanced discriminatory capacity enables more accurate identification of patients at high risk for IE [8].

Their main purpose of these scores is not to reduce workload alone, but rather to minimize the risk of missing IE cases while limiting unnecessary imaging. Yet, as with all diagnostic tools, their value depends on the balance between avoiding missed diagnoses and ensuring efficient use of resources. In this study, we validated the NOVA and DENOVA scores in a multicenter Swiss cohort and proposed a modified score, DENOVi, aimed at improving the balance between safely ruling out IE and reducing the number of unnecessary cardiac imaging examinations.

## METHODS

This retrospective study was conducted at Lausanne University Hospital (CHUV) and University Hospital Zurich (USZ) in Switzerland between January 2015 and June 2024. It included 3 distinct cohorts: (1) the CHUV bacteremia cohort, with patients retrospectively enrolled from January 2015 to December 2021, (2) the CHUV suspected IE cohort, with prospectively enrolled from January 2022 to June 2024, and (3) the USZ IE cohort, with retrospective inclusion from January 2015 to December 2017 and prospective inclusion from January 2018 to June 2024. The study was approved by the relevant Swiss ethics committees (CER-VD 2021-02516, CER-VD 2017-02137, KEK-2014-0461; BASEC-2017-01140). Suspected IE was defined as the combination of blood cultures being drawn and echocardiography performed specifically to investigate IE.

Inclusion criteria were: adult patients (≥18 years old), at least 1 blood culture positive for Enterococcus spp., and no documented refusal of data use. Exclusion criteria consisted of patients with incomplete medical records, such as those transferred to other hospitals at the onset of infection without available follow-up data.

Data on demographics, clinical presentation, imaging, microbiology, surgery, and pathology were extracted from patient's electronic health records. At both institutions, infectious diseases (ID) consultants were notified of all positive blood cultures once the organism was identified. Although ID consultation for enterococcal bacteremia was not mandatory and was provided at the discretion of the attending physician, it became obligatory whenever IE was suspected [15].

In both centers, blood cultures were incubated using the BacT/ALERT System (bioMerieux, Marcy l'Etoile, France). Species identification was performed using matrix-assisted laser desorption-ionization time of flight mass spectrometry (Bruker Daltonics, Bremen, Germany).

The date of the first positive blood culture was defined as the onset of infection. A new episode was recorded if more than 30 days had passed since the completion of antimicrobial therapy for the previous bacteremia. Classification of bacteremia cases as community-acquired, healthcare-associated, or nosocomial followed the criteria established by Friedman et al. [16]. The site of infection site was determined by the ID consultant, based on a comprehensive assessment of clinical, radiological, microbiological, and surgical findings.

To identify high-risk cases of IE, the adapted NOVA (≥4 points) was applied to all episodes [13], and the DENOVA (≥3 points) score was used for episodes with *E. faecalis* bacteremia (Supplementary Table 1) [11]. In addition, the DENOVA score was evaluated across all enterococcal bacteremia episodes using the same threshold. Recognizing the overlap between valve disease (V) and murmur (A) components of the score, we developed a modified score, named DENOVi by removing the A component [11], and updating the V component to include the presence of cardiac implantable electronic devices (Vi). Since the number of variables in the new score was reduced, we set the threshold for defining a high-risk episode at ≥2 points, as clinical judgment suggests that the presence of at least 2 variables should prompt cardiac imaging studies. Cases were further classified as rejected, possible, or definite IE according to the 2023 International Society of Cardiovascular Infectious Diseases (ISCVID) Duke criteria [17].

We used 2 reference standards: (1) the diagnosis made by the Endocarditis Team of each institution (since January 2018) or by expert clinicians prior to that date (CHUV: MPO, PM; USZ: BH, MVH) who served the role of adjudicating IE cases, and (2) the diagnosis based on the 2023 ISCVID Duke criteria [17].

The primary outcome was the negative likelihood ratio (NLR), reflecting the ability of the scores to safely rule out disease. Secondary outcomes included the proportion of echocardiograms warranted if the score was applied and the positive likelihood ratio (PLR).

Data analysis was performed using SPSS version 26.0 (SPSS, Chicago, IL, USA). Categorical variables were compared using the χ^2^ or Fisher exact test, while continuous variables were analyzed with the Mann–Whitney U test. The performance of the NOVA, DENOVA, and the newly developed DENOVi scores in identifying episodes at high risk for IE was evaluated by measuring the level of agreement between each score's high-risk classification and 2 reference standards. Sensitivity, specificity, positive and negative predictive values (PPV and NPV), PLR, NLR, and overall accuracy were calculated, each with corresponding 95% confidence intervals (CI). All tests were two-tailed, and a *P*-value of <.05 was considered statistically significant.

## RESULTS

**Figure 1.**
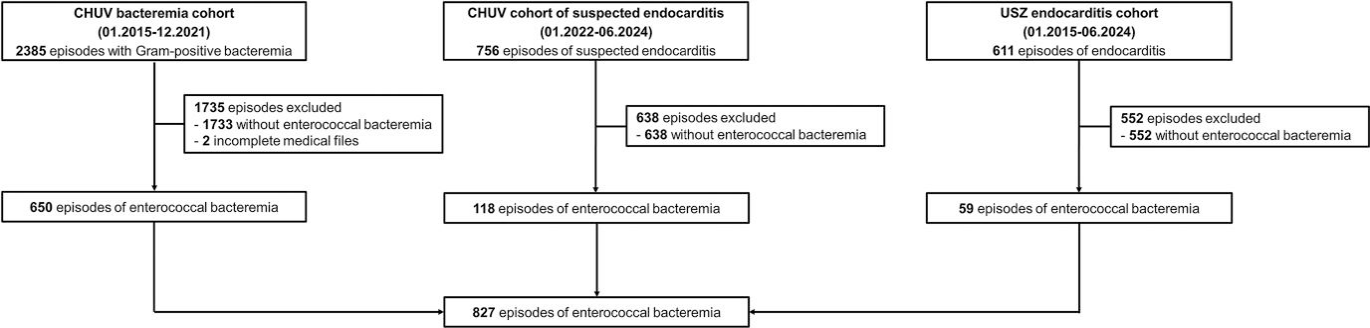
Flowchart of included episodes. CHUV: Lausanne University Hospital, USZ: University Hospital Zurich.

A total of 827 episodes of enterococcal bacteremia were included from the 3 cohorts (Figure 1 Of these, 480 (58%) were caused by *E. faecalis* and 326 (39%) by *E. faecium* (Table 1). Cardiac imaging was performed in 484 (58%) episodes. Specifically, transthoracic echocardiography (TTE) was performed in 442 (53%), transesophageal echocardiography (TEE) in 178 (22%), [18F]fluorodeoxyglucose positron emission tomography/computed tomography ([18F]FDG PET/CT) in 79 (10%), and cardiac CT in 13 (2%) episodes. Among the 442 episodes in which TTE was performed initially, 139 (31%) were subsequently followed by TEE. In 39 episodes, TEE was chosen as the first imaging modality. The reasons for upfront TEE varied: the majority of patients were already intubated due to severe illness or undergoing surgery for source control (25 episodes, 64%); in 7 episodes (18%), recurrent bacteremia with the same species occurred and the TTE performed during the initial episode was of poor quality; in 3 episodes (8%), suboptimal TTE imaging was anticipated due to obesity or thoracic wall deformities; and in 4 episodes (10%), the rationale was not documented.

**Table 1.** Comparison of Episodes With or Without Infective Endocarditis Among 827 Episodes With Enterococcal Bacteremia

	No Infective Endocarditis (*n* = 655)	Infective Endocarditis (*n* = 172)	*P*
Demographics			
Male sex, *n* (%)	455 (70)	128 (74)	.223
Age, median y (IQR)	70 (59–78)	70 (58–79)	.630
Setting of infection onset			<.001^a^
Community-acquired, *n* (%)	110 (17)	115 (67)	
Healthcare-associated, *n* (%)	105 (16)	31 (18)	
Nosocomial, *n* (%)	440 (67)	26 (15)	
Cardiac predisposing factors			
Intravenous drug use	9 (1)	20 (12)	<.001
Valve disease			
Prior endocarditis, *n* (%)	14 (2)	28 (16)	<.001
Native valve disease, *n* (%)	39 (6)	48 (28)	<.001
Prosthetic valve/transcatheter aortic valve replacement, *n* (%)	39 (6)	75 (44)	<.001
Cardiac implantable electronic device, *n* (%)	49 (8)	46 (27)	<.001
Microbiological data			
Species			
*E. faecalis*, *n* (%)	327 (50)	153 (89)	<.001
*E. faecium*, *n* (%)	308 (47)	18 (10)	<.001
Other enterococci, *n* (%)	49 (8)	3 (2)	.004
Two or more positive initial blood cultures positive, *n* (%)	353 (54)	154 (90)	<.001
Persistent enterococcal bacteremia (≥48 h), *n* (%)	59 (9)	32 (19)	.001
Polymicrobial bloodstream infection, *n* (%)	242 (37)	13 (8)	<.001
Initially unknown origin of bacteremia, *n* (%)	212 (32)	169 (98)	<.001
Infection data			
Duration of systemic symptoms, median days (IQR)	1 (1–1)	7 (2–27)	<.001
Duration of systemic symptoms ≥7 d, *n* (%)	25 (4)	99 (58)	<.001
Fever, *n* (%)	555 (85)	139 (81)	.243
Sepsis, *n* (%)	309 (47)	56 (33)	.001
Heart murmur, *n* (%)	78 (12)	95 (55)	<.001
Embolic events (within 72 h of first blood cultures), *n* (%)	15 (2)	54 (31)	<.001
Cerebral embolic events, *n* (%)	6 (.9)	29 (17)	<.001
Noncerebral embolic events, *n* (%)	10 (2)	38 (21)	<.001
Immunological phenomena, *n* (%)	0 (0)	8 (5)	<.001
Type of infection			
Unknown focus, *n* (%)	42 (6)	0 (0)	<.001
Catheter-related, *n* (%)	133 (20)	3 (2)	<.001
Urinary tract infection, *n* (%)	102 (16)	0 (0)	<.001
Abdominal infection, *n* (%)	307 (47)	3 (2)	<.001
Bone and joint infection, *n* (%)	24 (4)	17(10)	.002
Other infectious foci, *n* (%)	61 (9)	8 (5)	.062

Abbreviation: IQR, interquartile range.

^a^Comparison between nosocomial bacteremias and both community and healthcare-associated.

IE was diagnosed in 172 (21%) episodes, with 82% classified as definite and 18% as possible IE by the Endocarditis Teams. Supplementary Table 2 describes the 15 discordant episodes between the 2 reference standards. Recurrence of bacteremia with the same enterococcal species within 1 year was observed in 49 episodes (6%). Of these, 8 patients with initial IE experienced a subsequent IE episode, while 3 patients with an initial non-IE episode developed new IE within the year. Supplementary Table 3 provides a detailed summary of the enterococcal species, clinical diagnoses, and cardiac imaging findings for both the initial and recurrent episodes.

Among all 827 episodes of enterococcal bacteremia, 626 (76%) were categorized as high-risk by the adapted NOVA score (Table 2). Of the 480 *E. faecalis* bacteremia episodes, the DENOVA score classified 176 (37%) as high-risk. Applying DENOVA score to all enterococcal bacteremia episodes classified 211/827 (26%) as high-risk. The newly developed DENOVi score identified 351 (42%) as high-risk when applied to the full cohort.

**Table 2.** Classifications Based on the Adapted NOVA, DENOVA, and New DENOVi Scores and 3 Versions of the Duke Clinical Criteria

Adapted NOVA score (all enterococci)	*n* = 655	*n* = 172
NOVA-N (number of positive blood cultures, +5 points), *n* (%)	353 (54)	154 (90)
NOVA-O (unknown origin of bacteremia, +4 points), *n* (%)	212 (32)	167 (97)
NOVA-V (valve disease, +2 points), *n* (%)	81 (12)	113 (66)
NOVA-A (auscultation of a heart murmur, +1 point), *n* (%)	78 (12)	95 (55)
Final adapted NOVA score (points), median (IQR)	5 (0–6)	11 (10–12)
Adapted NOVA score ≥4 points, *n* (%)	456 (70)	170 (100)
DENOVA score (*E. faecalis*)	*n* = 327	*n* = 153
DENOVA-D (duration of symptoms ≥7 d, +1 point), *n* (%)	18 (6)	91 (60)
DENOVA-E (embolization, +1 point), *n* (%)	6 (2)	50 (33)
DENOVA-N (number of positive cultures ≥2, +1 point), *n* (%)	168 (51)	137 (90)
DENOVA-O (origin of infection unknown, +1 point), *n* (%)	122 (37)	150 (98)
DENOVA-V (valve disease, +1 point), *n* (%)	47 (14)	100 (65)
DENOVA-A (auscultation of murmur, +1 point), *n* (%)	49 (15)	88 (58)
Final DENOVA score (points), median (IQR)	1 (1–2)	4 (3–5)
DENOVA score ≥3 points, *n* (%)	35 (11)	141 (92)
DENOVA score (all enterococci)	*n* = 655	*n* = 172
DENOVA-D (duration of symptoms ≥7 d, +1 point), *n* (%)	25 (4)	99 (58)
DENOVA-E (embolization, +1 point), *n* (%)	15 (2)	54 (31)
DENOVA-N (number of positive cultures ≥2, +1 point), *n* (%)	353 (54)	154 (90)
DENOVA-O (origin of infection unknown, +1 point), *n* (%)	212 (32)	167 (97)
DENOVA-V (valve disease, +1 point), *n* (%)	81 (12)	113 (66)
DENOVA-A (auscultation of murmur, +1 point), *n* (%)	78 (12)	95 (55)
Final DENOVA score (points), median (IQR)	1 (1–2)	4 (3–5)
DENOVA score ≥3 points, *n* (%)	55 (8)	156 (91)
DENOVi score (all enterococci)	*n* = 655	*n* = 172
DENOVi-Vi (Valve disease, intracardiac electronic devices, +1 point), *n* (%)	122 (19)	122 (71)
Final DENOVi score (points), median (IQR)	1 (0–2)	4 (3–4)
DENOVi score ≥2 points, *n* (%)	184 (28)	167 (97)

Table 3 summarizes the diagnostic performance of the adapted NOVA, DENOVA, and DENOVi scores. The adapted NOVA classified 626/827 (76%) episodes as high risk, representing an absolute increase of 18% in the proportion of echocardiograms required compared with the 58% performed based on clinical evaluation alone. Two (1%) IE episodes were incorrectly classified as low risk, resulting in a NLR of 0.04 (95% CI .01–.15) and a PLR of 1.42 (1.35–1.50). The DENOVA score, when applied to *E. faecalis* bacteremia only, classified 176/335 (37%) episodes as high risk, with 12 (8%) IE episodes being incorrectly classified as low risk. The NLR was 0.09 (0.05–0.15) and the PLR 8.61 (6.27–11.8). When DENOVA was applied to all enterococcal species, 211/827 (26%) were classified as high risk, representing an absolute increase of 32% in the proportion of echocardiograms required compared with clinical evaluation alone. DENOVA misclassified 16 (9%) IE episodes as low risk, resulting to a NLR of 0.10 (0.06–0.16) and a PLR 10.8 (8.35–14.0). The DENOVi score identified 351/827 (42%) episodes as high risk, representing an absolute increase of 16% in the proportion of echocardiograms required compared with clinical evaluation alone. Only 5 (3%) IE episodes were incorrectly classified as low risk, resulting in a NLR of 0.04 (0.02–0.10) and a PLR of 3.46 (3.05–3.92). The performance of the scores, using the 2023 ISCVID Duke criteria as the reference standard, is shown in Table 4. Supplementary Table 4 summarizes the performance of the 3 scores in monomicrobial enterococcal bacteremia with the reference standard being the diagnosis of the Endocarditis Team.

**Table 3.** Performance of the Adapted NOVA, DENOVA, and New DENOVi Scores in Identifying Patients at High-risk for Infective Endocarditis Among 827 Episodes of Enterococcal Bacteremia With the Reference Standard Being the Diagnosis of the Endocarditis Team

	Episodes Classified As High Risk *N* (%)	Sensitivity % (95% CI)	Specificity % (95% CI)	PPV % (95% CI)	NPV % (95% CI)	PLR % (95% CI)	NLR % (95% CI)	Accuracy % (95% CI)
Adapted NOVA score ≥4 points	626 (76)	99 (96–100)	30 (27–34)	27 (26–28)	99 (96–100)	1.42 (1.35–1.50)	.04 (.01–.15)	45 (41–48)
DENOVA score ≥3 points (*E. faecalis*; *n* = 335)	176 (37)	92 (87–96)	89 (85–92)	80 (75–85)	96 (93–98)	8.61 (6.27–11.8)	.09 (.05–.15)	90 (87–93)
DENOVA score ≥3 points	211 (26)	91 (85–95)	92 (89–94)	74 (69–79)	97 (96–98)	10.8 (8.35–14.0)	.10 (.06–.16)	91 (89–93)
DENOVi score ≥2 points	351 (42)	97 (93–99)	72 (69–76)	48 (45–51)	99 (98–100)	3.46 (3.05–3.92)	.04 (.02–.10)	77 (74–80)

Abbreviations: NLR, negative likelihood ratio; NPV, negative predictive value; PLR, positive likelihood ratio; PPV, positive predictive value.

**Table 4.** Performance of the Adapted NOVA, DENOVA, and New DENOVi Scores in Identifying Patients at High-risk for Infective Endocarditis Among 827 Episodes of Enterococcal Bacteremia With the Reference Standard Being the 2023 ISCVID Duke Criteria

	Episodes Classified As High Risk *N* (%)	Sensitivity % (95% CI)	Specificity % (95% CI)	PPV % (95% CI)	NPV % (95% CI)	PLR % (95% CI)	NLR % (95% CI)	Accuracy % (95% CI)
Adapted NOVA score ≥4 points	626 (76)	98 (95–99)	31 (27–34)	29 (28–30)	98 (95–99)	1.41 (1.34–1.49)	.07 (.03–.19)	46 (42–49)
DENOVA score ≥3 points (*E. faecalis*; *n* = 335)	176 (37)	89 (83–93)	91 (87–94)	83 (78–87)	94 (91–96)	9.38 (6.64–13.2)	.12 (.08–.19)	90 (90–93)
DENOVA score ≥3 points	211 (26)	87 (81–91)	92 (90–94)	76 (71–81)	96 (94–97)	11.1 (8.45–14.6)	.15 (.10–.21)	91 (89–93)
DENOVi score ≥2 points	351 (42)	94 (89–97)	73 (69–76)	50 (47–53)	98 (96–99)	3.43 (3.01–3.91)	.08 (.05–.14)	77 (74–80)

Abbreviations: ISCVID, International Society of Cardiovascular Infectious Diseases; NLR, negative likelihood ratio; NPV, negative predictive value; PLR, positive likelihood ratio; PPV, positive predictive value.

In the CHUV's bacteremia cohort (*n* = 650), IE was diagnosed in 47 episodes (7%) by the Endocarditis Team and in 57 episodes (9%) by the 2023 ISCVID Duke criteria (47 definite IE, 10 possible IE). Supplementary Table 5 shows the performance of the respective scores in the 650 episodes of enterococcal bacteremia from CHUV.

## DISCUSSION

This large dual-center study highlights the strengths and weaknesses of predictive scores for IE among patients with enterococcal bacteremia. Both the adapted NOVA and DENOVi scores had lower NLRs compared with the DENOVA score, while the DENOVi score showed a higher PLR and required fewer echocardiograms than the adapted NOVA.

The adapted NOVA score yielded a sensitivity of 99%, an NLR of 0.04, and an NPV of 99%. These metrics reaffirm the value of NOVA in safely ruling out disease [12, 13]. Importantly, only 2 IE episodes (1%) would have been missed if applied to our population. This extremely low false omission rate makes NOVA a reassuring tool for clinicians seeking to avoid diagnostic errors. However, the NOVA score has several limitations [10, 13]. Notably, the adapted NOVA score classifies a large majority of patients as high risk (76%). This translates into a significant increase in echocardiograms compared with clinical judgment alone. For hospitals with already high imaging volumes, this expansion could result in unsustainable strain on cardiology and infectious disease services. Additionally, although the NOVA score consists of 4 components, only 2—namely, the number of positive blood cultures and unknown origin of bacteremia—determine the risk classification. Patients meeting either criterion are automatically classified as high-risk, while those without are considered low-risk, even if both remaining criteria (pre-existing valve disease and auscultation of a heart murmur) are present. These latter components together contribute only 3 points, which is insufficient to reach the ≥4-point threshold for high-risk classification [10]. A further limitation of the NOVA score is the exclusion of embolic events, which are among the strongest predictors of IE [10, 18]. In addition, embolic events, which are strong independent predictors of IE, are not captured, leaving the score blind to an important subset of high-risk patients.

The 2023 ESC guidelines recommend the DENOVA score over the NOVA score due to its superior discriminatory power [8]. The DENOVA score was designed to refine discrimination and improve specificity, especially among patients with *E. faecalis* bacteremia. In our cohort, DENOVA demonstrated high specificity (89% for *E. faecalis* and 92% when applied to all enterococci) and excellent PLRs (8.61 and 10.8, respectively), confirming its strong capacity to “rule in” disease. The sensitivity was slightly lower than the sensitivity reported in previous studies, which ranged from 95% to 100% [11, 12, 14]. This observed discrepancy in sensitivity may be explained by 2 factors: first, our study included episodes of polymicrobial *E. faecalis* bacteremia, which were not considered in previous studies; however in the analysis of episodes with monomicrobial *E. faecalis* bacteremia the sensitivity was 94%. Second, prior evaluations of the DENOVA score relied on the 2015 Duke-ESC criteria as the reference standard. These criteria share several variables with the DENOVA score, such as 2 positive blood culture sets, embolic events, cardiac predisposing conditions, potentially inflating diagnostic accuracy due to incorporation bias [11, 12, 14, 19]. To address this, we used 2 distinct reference standards: the clinical diagnosis by a multidisciplinary Endocarditis Team and the diagnosis based on the 2023 ISCVID Duke criteria. In addition, we expanded the assessment of the DENOVA score to include all enterococcal species, not just *E. faecalis* as originally intended [11]. The score showed comparable sensitivity and slightly higher specificity in this broader context, supporting its potential applicability to all enterococcal bacteremias. While the DENOVA score had the highest PLR and the greatest absolute reduction in warranted echocardiograms compared with the other scores, it also had the highest NLR. In clinical practice, however, the primary priority of predictive scores should be their ability to safely rule out IE rather than merely reducing the number of echocardiograms or ruling in disease. Consequently, these findings challenge the 2023 ESC guidelines, which recommend the use of the DENOVA score [8].

To overcome the specific shortcomings of the original DENOVA score, we developed a modified version termed DENOVi. This revision incorporated intracardiac electronic devices into the valve disease category and eliminated the auscultation of a murmur, a parameter considered unreliable due to overlap with valve disease and high interobserver variability [11, 20, 21]. By removing this subjective element and recognizing the increasing importance of device-associated infections, the score was modernized to reflect current clinical realities. A new cutoff of ≥2 points was introduced to align with the reduced number of variables and maintain adequate discriminatory power. With this adaptation, performance improved: sensitivity rose to 97% compared with 91% for DENOVA, and the NPV reached 99%. The NLR was 0.04, identical to that of the adapted NOVA score, underscoring DENOVi's strength as a rule-out tool. These results suggest that patients with DENOVi scores of 0 or 1, in the absence of strong clinical suspicion, may not require echocardiographic evaluation. Furthermore, DENOVi demonstrated a higher PLR, enhancing its value for risk stratification. Moreover, DENOVi would have reduced the number of echocardiograms performed, whereas the adapted NOVA would have increased them. Overall, DENOVi appeared equally effective to adapted NOVA in safely ruling out IE, while requiring fewer echocardiograms, thus offering stronger discriminatory performance. Nonetheless, external validation in larger and more diverse populations is necessary before the score can be adopted in clinical practice. If confirmed, DENOVi could serve as a valuable complement to clinician judgment, helping optimize use of resources while minimizing unnecessary diagnostic procedures.

This study has several limitations. First, it was retrospective and restricted to 2 university hospitals within 1 country, limiting generalizability. Second, inclusion of cases from both the CHUV's suspected IE cohort and the USZ IE cohort may have artificially inflated sensitivity estimates. However, it was intended to improve the precision and stability of our diagnostic performance estimates, particularly for sensitivity analyses. Misclassification of IE as non-IE carries greater clinical and methodological consequences than the converse; thus, expanding the pool of adjudicated IE cases was essential for a balanced evaluation. To address this, we conducted a separate analysis confined to CHUV's bacteremia cohort. Third, cardiac imaging was not performed in 42% of episodes. Most of these patients received short antibiotic courses (<14 days) and showed no signs of IE within 120 days, making undiagnosed cases unlikely but not impossible [22]. Fourth, reliance on the Endocarditis Team's clinical diagnosis as the reference standard introduces potential misclassification bias. To mitigate this, we also applied the 2023 ISCVID Duke criteria for parallel validation. Fifth, although several patients experienced a recurrence of bacteremia and/or IE within 1 year of the initial episode, the absence of molecular comparison of the strains meant that we could not determine whether these represented relapses or reinfections [8, 23]. Nevertheless, each recurrent episode was evaluated individually, taking into account additional risk factors for IE (Supplementary Table 3). Finally, predictive values are strongly influenced by disease prevalence. Although our NPVs exceeded 90% for all 3 scores, a proportion of IE cases would still have been misclassified as low risk. These findings underscore the unavoidable tradeoff inherent in score-based strategies: reducing unnecessary echocardiograms must be balanced against the greater priority of avoiding missed IE cases. Thus, while prediction scores may support clinical decision-making, they should not be used in isolation, particularly in high-prevalence settings.

In summary, both adapted NOVA and DENOVi demonstrated strong rule-out capacity among episodes with enterococcal bacteremia, clearly outperforming DENOVA. However, the adapted NOVA tended to overestimate risk, leading to more echocardiograms, while DENOVi resulted to a decrease in warranted echocardiograms. Thus, DENOVi offered the most balanced diagnostic performance, reducing missed IE cases without overburdening resources. If validated externally, DENOVi could become a useful adjunct to clinical judgment in patients with enterococcal bacteremia, enhancing efficiency and supporting judicious use of cardiac imaging.

## Supplementary Material

ofaf796_Supplementary_Data

## Supplementary Data

Supplementary materials are available at *Open Forum Infectious Diseases* online. Consisting of data provided by the authors to benefit the reader, the posted materials are not copyedited and are the sole responsibility of the authors, so questions or comments should be addressed to the corresponding author.

## Notes

***Author Contributions***. M.P.O. and B.H. conceived the idea. V.Z., N.F., J.E., P.M., G.T., M.F., L.S., N.I., G.C., L.N., M.K., M.V.H., O.D., B.G., and M.P.O. collected the patients’ data. M.P.O., B.L., and B.H. supervised the project. B.L. and M.P.O. performed the analysis and interpreted the results. M.P.O. wrote the manuscript. All authors contributed to manuscript revision and read and approved the submitted version.

***Financial support.*** This work was supported by the Swiss National Science Foundation (SNSF) grant #32003B_219351/1 (to B.H. and M.P.O.), the Swiss Heart Foundation #FF24013 (to M.P.O., B.H., and P.M.), and the Vaudois Foundation for Interventional Cardiology grant (to M.P.O. and P.M.). The funders had no role in study design, data collection and analysis, decision to publish, or preparation of the manuscript. There are no relationships with industry.

***Patient Consent Statement***. The study received approval from the relevant Swiss ethics committees (CER-VD 2021-02516, CER-VD 2017-02137, KEK-2014-0461; BASEC-2017-01140). For the prospective parts, all participants signed the informed consent to participate. For the retrospective parts, the ethic committee waived the need of informed consent to participate; however, patients were excluded if they had previously refused to permit the use of their data for research purposes.
