# Supplementary material for: Prediction Scores Identifying Patients at High Risk of Endocarditis in Enterococcal Bacteremia
Source: Open Forum Infect Dis. 2025 Dec 23;13(1):ofaf796. doi: 10.1093/ofid/ofaf796 (PMC12784196; doi:10.1093/ofid/ofaf796)
Supplement: ofaf796_Supplementary_Data [file ofaf796_supplementary_data.pdf]

Supplementary Table 1. Overview of adapted NOVA, DENOVA and DENOVI scores

**Adapted NOVA score**		**DENOVA score**		**DENOVi score**	
Population: all enterococci		Population: *Enterococcus faecalis*		Population: all enterococci	
**Items**	**Points**	**Items**	**Points**	**Items**	**Points**
		**D:** Duration of symptoms ≥7 days	1	**D:** Duration of symptoms ≥7 days	1
		**E:** Embolization	1	**E:** Embolization	1
**N:** Number of positive blood cultures[a]	5	**N:** Number of positive blood cultures[a]	1	**N:** Number of positive blood cultures[a]	1
**O:** Unknown origin of bacteremia	4	**O:** Unknown origin of bacteremia	1	**O:** Unknown origin of bacteremia	1
**V:** Valve disease[b]	2	**V:** Valve disease[b]	1	**Vi:** Valve disease[b], intracardiac electronic devices[c]	1
**A:** Auscultation of a heart murmur	1	**A:** Auscultation of a heart murmur	1		
Cutoff: ≥4 points		Cutoff: ≥3 points		Cutoff: ≥2 points	

[a]Number of positive blood cultures refers to two out of two positive blood culture sets, three out of three positive sets, or the majority of sets if more than three were drawn.

[b]Valve disease refers to native valve disease (classified as moderate or high risk for infective endocarditis), previous infective endocarditis, or the presence of a prosthetic valve.

[b]Intracardiac electronic devices refer to cardiac implantable electronic devices

Supplementary Table 2. Discordant episodes between the two reference standards

	Endocarditis Team diagnosis	Microbiologic criterion	Imaging criterion	Predisposition	Fever	Vascular or immunologic phenomena	Other information
1	No IE	*E. faecalis* (2 bcs)		Prosthetic valve	Y	Janeway lesions	Septic shock due to urinary-tract infection; petechias mistaken for Janeway lesions; TEE negative for IE; antibiotic treatment for less than 14 days; no episode of bacteremia/IE in the subsequent 120 days
2	No IE	*E. faecalis* (2 bcs)	Significant new valvular regurgitation[a]	Moderate valvular regurgitation[a]	Y		Nosocomial bacteremia; abdominal infection; TEE negative for IE; antibiotic treatment for less than 14 days; no episode of bacteremia/IE in the subsequent 120 days
3	No IE	*E. faecalis* (2 bcs)<br>E. coli (2 bcs)	Significant new valvular regurgitation[a]	Moderate valvular regurgitation[a]	Y		Nosocomial bacteremia; abdominal infection; TEE negative for IE; antibiotic treatment for less than 14 days; no episode of bacteremia/IE in the subsequent 120 days
4	No IE	*E. faecalis* (2 bcs)	Significant new valvular regurgitation		Y		Nosocomial bacteremia; catheter-related bacteremia; TEE negative for IE; antibiotic treatment for less than 14 days; no episode of bacteremia/IE in the subsequent 42 days
5	No IE	*E. faecalis* (2 bcs)<br>E. coli (2 bcs)		Moderate valvular regurgitation	Y	Cerebral emboli	Nosocomial bacteremia; abdominal infection; TEE negative for IE; ischemic stroke at day 11 due to atrial fibrillation
6	No IE	*E. faecalis* (2 bcs)	Significant new valvular regurgitation		Y		Nosocomial bacteremia; urinary-tract infection

7	No IE	*E. faecalis* (2 bcs)	Significant new valvular regurgitation		Y	Nosocomial bacteremia; urinary-tract infection; antibiotic treatment for less than 14 days; no episode of bacteremia/IE in the subsequent 120 days
8	No IE	*E. faecalis* (2 bcs)<br>*E. coli* (2 bcs)	Significant new valvular regurgitation		Y	Urinary-tract infection; antibiotic treatment for less than 14 days; no episode of bacteremia/IE in the subsequent 120 days
9	No IE	*E. faecium* (3 bcs)	Significant new valvular regurgitation		Y	Nosocomial bacteremia; abdominal infection; antibiotic treatment for less than 14 days; no episode of bacteremia/IE in the subsequent 120 days
10	No IE	*E. raffinosus* (2 bcs)<br>*K. pneumoniae* (2 bcs)	Significant new valvular regurgitation		N	Abdominal infection; antibiotic treatment for less than 14 days; no episode of bacteremia/IE in the subsequent 120 days
11	No IE	*E. faecalis* (3 bcs)	Significant new valvular regurgitation	Prior IE	N	Catheter-related bacteremia; TEE and [18F]FDG PET/CT negative for IE; antibiotic treatment for less than 14 days; no episode of bacteremia/IE in the subsequent 120 days
12	No IE	*E. faecalis* (3 bcs)	Significant new valvular regurgitation	CIED	N	Bacteremia of unknown origin; TEE and [18F]FDG PET/CT negative for IE; antibiotic treatment for less than 14 days; no episode of bacteremia/IE in the subsequent 22 days
13	No IE	*E. faecalis* (2 bcs)	Significant new valvular regurgitation	CIED	Y	Nosocomial bacteremia; TEE and [18F]FDG PET/CT negative for IE; antibiotic treatment for less than 14 days; no episode of bacteremia/IE in the subsequent 120 days

14	No IE	*E. faecium* (3 bcs)		CIED	Y	Pulmonary emboli	Catheter-related infection with septic deep vein thrombosis; [18F]FDG PET/CT negative for IE; antibiotic treatment for less than 14 days; no episode of bacteremia/IE in the subsequent 29 days
15	IE	*E. faecalis* (1 bcs)	Valve leaflet thickening		Y		Bacteremia of unknown origin; only one blood culture set was dawn before antimicrobial treatment initiation

[18F]FDG PET/CT: [18F]fluorodeoxyglucose positron emission tomography/computed tomography; bcs: blood culture set; CIED: cardiac implantable electronic device; IE: infective endocarditis; N: no; TEE: transesophageal echocardiography; Y: yes

[a]the significant new valvular regurgitation (imaging criterion) appeared in a different valve from the moderate valvular regurgitation previously observed (predisposition criterion)

Supplementary Table 3. Diagnoses of episodes with recurrent bacteremia caused by the same enterococcal species within one year of the initial episode

		**initial episode**		**Subsequent episode**	
	**Species**	**Diagnosis**	**Cardiac imaging**	**Diagnosis**	**Cardiac imaging**
1	*E. faecalis*	Infective endocarditis	TTE, TEE, [18F]FDG PET/CT	Infective endocarditis	TTE, [18F]FDG PET/CT
2	*E. faecalis*	Infective endocarditis	TTE	Infective endocarditis	TTE, TEE
3	*E. faecalis*	Infective endocarditis	TTE	Infective endocarditis	TEE, [18F]FDG PET/CT
4	*E. faecalis*	Infective endocarditis	TTE	Infective endocarditis	TTE, TEE
5	*E. faecalis*	Infective endocarditis	TTE, TEE	Infective endocarditis	TTE, TEE
6	*E. faecalis*	Infective endocarditis	TTE, TEE, cardiac CT	Infective endocarditis	TTE
7	*E. faecalis*	Infective endocarditis	TTE, TEE	Infective endocarditis	TTE, TEE
8	*E. faecalis*	Infective endocarditis	TTE, TEE	Infective endocarditis	TEE
9	*E. faecalis*	Infective endocarditis	TTE, TEE, [18F]FDG PET/CT	Catheter-related	TTE, TEE, [18F]FDG PET/CT
10	*E. faecalis*	Unknown origin	TTE, TEE, [18F]FDG PET/CT	Infective endocarditis	TTE, TEE
11	*E. faecalis*	Unknown origin	TTE, TEE	Infective endocarditis	TTE, [18F]FDG PET/CT
12	*E. faecalis*	Unknown origin	TTE	Bone and joint	TEE
13	*E. faecalis*	Unknown origin	TTE, TEE	Unknown origin	TTE, TEE, [18F]FDG PET/CT
14	*E. faecalis*	Abdominal		Abdominal	TTE
15	*E. faecalis*	Abdominal	TTE	Abdominal	TTE, TEE
16	*E. faecalis*	Abdominal		Abdominal	TTE
17	*E. faecalis*	Urinary-tract		Urinary-tract	
18	*E. faecalis*	Urinary-tract		Urinary-tract	
19	*E. faecalis*	Urinary-tract	TTE, TEE, [18F]FDG PET/CT	Infective endocarditis	TTE, TEE

20	*E. faecalis*	Urinary-tract		Catheter-related	
21	*E. faecalis*	Urinary-tract		Bone and joint	TTE, TEE
22	*E. faecalis*	Urinary-tract	TTE	Bone and joint	TTE, TEE
23	*E. faecalis*	Catheter-related		Catheter-related	
24	*E. faecalis*	Catheter-related	TTE, TEE, [18F]FDG PET/CT	Unknown origin	TTE, TEE, [18F]FDG PET/CT
25	*E. faecalis*	Catheter-related	TTE	Skin and soft tissue infection	TTE
26	*E. faecalis, E. faecium*	Abdominal	TTE	Abdominal	TTE
27	*E. faecium*	Abdominal		Abdominal	TTE
28	*E. faecium*	Abdominal	TTE	Abdominal	TTE
29	*E. faecium*	Abdominal		Abdominal	TTE
30	*E. faecium*	Abdominal	TTE	Abdominal	TTE
31	*E. faecium*	Abdominal		Abdominal	
32	*E. faecium*	Abdominal	TTE	Abdominal	TTE, TEE
33	*E. faecium*	Abdominal		Abdominal	
34	*E. faecium*	Abdominal		Abdominal	
35	*E. faecium*	Abdominal		Abdominal	TTE, TEE
36	*E. faecium*	Abdominal		Abdominal	
37	*E. faecium*	Abdominal		Abdominal	TTE
38	*E. faecium*	Abdominal	TTE	Abdominal	TTE
39	*E. faecium*	Abdominal		Abdominal	
40	*E. faecium*	Abdominal		Abdominal	
41	*E. faecium*	Abdominal	TTE	Catheter-related	TTE

42	*E. faecium*	Catheter-related	TTE	Catheter-related	TTE
43	*E. faecium*	Catheter-related	TTE	Catheter-related	TTE, TEE
44	*E. faecium*	Catheter-related		Catheter-related	TTE
45	*E. faecium*	Catheter-related		Portal vein septic thrombosis	TTE
46	*E. faecium*	Urinary-tract	TTE, TEE	Urinary-tract	TTE
47	*E. faecium*	Catheter-related	TTE	Abdominal	TEE, [18F]FDG PET/CT
48	*E. faecium*	Catheter-related	TTE	Bone and joint	TTE
49	*E. avium*	Abdominal	TTE	Catheter-related	TEE

[18F]FDG PET/CT: [18F]fluorodeoxyglucose positron emission tomography/computed tomography; TEE: transesophageal echocardiography; TTE transthoracic echocardiography

Supplementary Table 4. Performance of the adapted NOVA, DENOVA and new DENOVi scores in identifying patients at high-risk for infective endocarditis among 572 episodes of monomicrobial enterococcal bacteremia with the reference standard being the diagnosis of the Endocarditis Team

	**Episodes classified as high risk N (%)**	**Sensitivity % (95% CI)**	**Specificity % (95% CI)**	**PPV % (95% CI)**	**NPV % (95% CI)**	**PLR % (95% CI)**	**NLR % (95% CI)**	**Accuracy % (95% CI)**
Adapted NOVA score ≥4 points	448 (78)	100 (89-100)	30 (26-35)	35 (34-37)	100 (97-100)	1.43 (1.34-1.52)	0.00 (0.00-0.09)	49 (45-54)
DENOVA score ≥3 points (*E. faecalis*; n=325)	165 (51)	94 (88-97)	83 (77-88)	81 (76-86)	94 (90-97)	5.50 (3.98-7.60)	0.08 (0.04-0.14)	88 (84-91)
DENOVA score ≥3 points	194 (34)	94 (89-92)	89 (86-92)	77 (71-81)	97 (95-99)	8.60 (6.51-11.4)	0.07 (0.04-0.13)	90 (88-93)
DENOVi score ≥2 points	278 (49)	99 (96-100)	71 (66-75)	56 (53-60)	99 (97-100)	3.37 (2.90-3.92)	0.02 (0.00-0.07)	79 (75-82)

NLR: negative likelihood ratio; NPV: negative predictive value; PLR: positive likelihood ratio; PPV: positive predictive value

Supplementary Table 5. Performance of the adapted NOVA, DENOVA and new DENOVi scores in identifying patients at high-risk for infective endocarditis among 650 episodes of enterococcal bacteremia from the CHUV's bacteremia cohort with the reference standard being the diagnosis of the Endocarditis Team

	**Episodes classified as high risk N (%)**	**Sensitivity % (95% CI)**	**Specificity % (95% CI)**	**PPV % (95% CI)**	**NPV % (95% CI)**	**PLR % (95% CI)**	**NLR % (95% CI)**	**Accuracy % (95% CI)**
Adapted NOVA score ≥4 points	453 (70)	98 (89-100)	33 (29-36)	10 (10-11)	99 (97-100)	1.45 (1.35-1.55)	0.07 (0.01-0.46)	37 (34-41)
DENOVA score ≥3 points (*E. faecalis*; n=335)	61 (18)	93 (80-98)	92 (88-95)	62 (53-71)	99 (97-100)	11.9 (7.93-17.7)	0.08 (0.03-0.24)	92 (89-95)
DENOVA score ≥3 points	84 (13)	91 (80-98)	93 (91-95)	51 (44-59)	99 (98-100)	13.5 (9.89-18.3)	0.09 (0.04-0.23)	93 (91-95)
DENOVi score ≥2 points	351 (42)	94 (82-99)	74 (70-77)	22 (19-25)	99 (98-100)	3.60 (3.08-4.19)	0.09 (0.03-0.26)	75 (72-79)

NLR: negative likelihood ratio; NPV: negative predictive value; PLR: positive likelihood ratio; PPV: positive predictive value
